# Enhanced BMP-2/BMP-4 ratio in patients with peripheral spondyloarthritis and in cytokine- and stretch-stimulated mouse chondrocytes

**DOI:** 10.1186/s13075-020-02330-9

**Published:** 2020-10-12

**Authors:** Anne Briolay, Alaeddine El Jamal, Paul Arnolfo, Benoît Le Goff, Frédéric Blanchard, David Magne, Carole Bougault

**Affiliations:** 1 Univ Lyon, Univ Claude Bernard Lyon 1, CNRS UMR 5246, ICBMS, F-69622 Lyon, France; 2 INSERM UMR1238, Nantes University, Nantes, France; 3 Rheumatology Department, CHU Nantes, Nantes, France

**Keywords:** Spondyloarthritis, BMPs, Ossification, Stretch, Cytokine, Enthesis

## Abstract

**Background:**

Excessive bone formation in the entheses is one of the features of peripheral spondyloarthritis (SpA). Complex pathological mechanisms connecting inflammation, mechanical stress, and ossification are probably involved. We focused on bone morphogenetic protein (BMP)-2, -4, and -7 as possible mediators of this process.

**Methods:**

BMP-2, -4, and -7 concentration was measured by ELISA in synovial fluids (SFs) of SpA (*n* = 56) and osteoarthritic (*n* = 21) patients. Mouse organotypic ankle cultures were challenged by a pro-inflammatory cocktail. Mouse primary chondrocytes, osteoblasts, or tenocytes were treated with TNF-α, interleukin (IL)-17, or IL-22 and/or subjected to cyclic stretch, or with recombinant BMP-2 or -4.

**Results:**

In SpA SFs, if BMP-7 was barely detectable, BMP-2 concentration was higher and BMP-4 was lower than in osteoarthritic samples, so that BMP-2/BMP-4 ratio augmented 6.5 folds (*p* < 0.001). In SpA patients, TNF-α, IL-6, and IL-17 levels correlated this ratio (*n* = 21). *Bmp-2/Bmp-4* ratio was similarly enhanced by cytokine treatment in explant and cell cultures, at mRNA level. In particular, simultaneous application of TNF-α and cyclical stretch induced a 30-fold increase of the *Bmp-2*/*Bmp-4* ratio in chondrocytes (*p* = 0.027). Blockade of prostaglandin E_2_ and IL-6 production had almost no effect on the stretch-induced regulation of *Bmp-2* or *-4*. Osteoinductive effects of BMP-4, and to a lesser extend BMP-2, were identified on cultured chondrocytes and tenocytes.

**Conclusions:**

Our results first settle that BMP factors are locally deregulated in the SpA joint. An unexpected decrease in BMP-4 could be associated to an increase in BMP-2, possibly in response to mechanical and/or cytokine stimulations.

## Background

Spondyloarthropathy is a common rheumatic disease, with a worldwide prevalence estimated at between 0.2 and 1.6% [1]. Patients whose symptoms are principally peripheral rather than axial are grouped under the term peripheral spondyloarthritis (SpA), which includes psoriatic arthritis, reactive arthritis, and enteropathic arthritis, among others. Frequently involved joints include the hands, wrists, elbows, shoulders, knees, ankles, and feet. Excessive bone formation is observed in the entheses, the bony insertions of the tendons and ligaments, which can lead in advanced stages to ankylosis. The pain is typically relieved by nonsteroidal anti-inflammatory drugs (NSAIDs), anti-TNF agents, or anti-interleukin (IL)-17 antibodies. All of these treatments have beneficial effects on inflammatory lesions, and long-term control of inflammation appears critical to limit ectopic ossification [2, 3]. The hierarchy between inflammation, mechanical stress, and ossification mechanisms in SpA pathophysiology seems complex.

The enthesis is structured as an organ, where the tendons attach to the bone through fibrocartilage connections. The mechanism of enthesophyte formation in SpA is still unclear. The bony spurs probably develop both by endochondral and by intramembranous ossification, i.e., both by cartilage-to-bone transition process and by direct deposition of osteoid on the underlying bone [4]. The process of endochondral ossification is however considered prevalent [3, 5, 6]. In this case, within an enthesis, ossification could be initiated by the metaplasia of tendon cells into fibro-chondrocytes and then chondrocytes [5].

The causes of the ectopic and excessive deposition of bone mineralized matrix still remain incompletely understood [3]. On the one hand, enthesitis is a characteristic feature of SpA. The inflammation process is relatively well deciphered: in addition to the pleotropic role of TNF-α, after activation by IL-23, T cells can promote local inflammation in the enthesis through IL-17 and IL-22, in particular. But in most pathological contexts, bone inflammation leads to enhanced resorption and suppressed formation [7]. On the other hand, biomechanical stress is proposed to occupy a central place in SpA pathophysiology, but the precise mechanisms leading to a pathological response of the enthesis are still largely unknown. Mechanical stretch may trigger ossification by entheseal cells. Numerous data have already shown that mechanical strain can enhance bone formation activity in osteoblasts [8]. In comparison, little is known regarding the effect of mechanical strain on chondrocytes and tendon cells in terms of mineralization. A particular cyclic tensile strain has been shown to upregulate expression of hypertrophic markers in primary chondrocytes [9]; and cyclic stretch may also favor osteogenic orientation of ligament cells [10–12]. Biomechanical stress and inflammation are certainly interconnected. In particular, microdamages induced by mechanical stress can participate in inflammation. But also, inflammation-induced bone loss can change the distribution of forces in the joint, in turn causing new bone formation to stabilize the structure [2]. Overall, further research on the relationship between inflammatory cytokines, mechanical stress, growth factors, and target entheseal cells are still needed.

Bone morphogenetic proteins (BMPs) are known to play an essential role in promoting bone formation. They not only regulate the chondro- and osteoblasto-genesis during skeletal development, so as to recap the endochondral ossification, but also play a role in the preservation of bone homeostasis afterward, and enhance the bone fracture healing. To date, more than 20 BMP members have been characterized, belonging to the transforming growth factor beta (TGF-β) superfamily [13]. BMPs trigger cellular responses mainly through the Smad pathway, but also through the mitogen-activated protein kinase pathway. Activation of the canonical Smad pathway consists of R-Smads phosphorylation (including Smad1, 5, and 8) and dimerization with the common partner Smad4, before nuclear translocation to regulate gene transcription. Higher serum levels of BMP-2, -7 but also -4 were found in SpA patients, and BMP-2 and -4 levels had a significant correlation with spinal radiograph scores, and BMP-7 levels reflected radiographic damage [14, 15]. Recently, abnormal osteogenic differentiation of mesenchymal stem cells from patients with axial SpA was shown to be the consequence of an imbalance between BMP-2 and Noggin, a BMP antagonist [16]. In addition, genetic polymorphisms in a BMP receptor have been identified as a predisposition to the ossification of a spine ligament [17]. In this pathology, with clinical features close to SpA symptoms, the expression of BMP receptors is particularly high [18]. Besides, the over-expression of Noggin reduced the incidence and severity of the spontaneous SpA-like arthritis of male DBA/1 mice [6]. In this context, we hypothesized that BMP family members may stimulate the first steps of enthesis ossification in SpA and therefore constitute promising targets.

In this study, we investigated the possible deregulation in BMP factors in the joint of SpA patients. We focused on BMP-2, -4, and -7 which have already been found deregulated in SpA patients. To determine if levels of BMP were locally impaired in the entheseal environment, BMP contents were assessed in SpA patients’ synovial fluids. Using organotypic and cellular models, we then aimed to determine which cells in the enthesis might produce BMPs, and whether stretch and/or cytokines might be responsible for a local imbalance in BMP content.

## Methods

### Human synovial fluids

Synovial fluids (SFs) were obtained from 21 patients with osteoarthritis (OA) and 56 patients with peripheral spondyloarthritis (SpA). SFs were sampled during an arthrocentesis. Cells were removed by centrifugation before storage at − 80 °C. The study was approved by the local ethics committee and by the French Research Ministry (N°2008–402). All enrolled patients have given their formal consent. OA was diagnosed according to the EULAR criteria [19]. Patients with OA included 12 males (57%) and 9 females, with a mean age of 63 ± 15 years old (mean ± SD). All OA SFs were typically non-inflammatory, with less than 500 elements per mm^3^. SpA patients satisfied the Assessment of SpondyloArthritis International Society (ASAS) criteria for peripheral SpA [20]. SpA patients’ characteristics are detailed in Table 1. C-reactive protein (CRP) serum concentration was measured for 34 patients with SpA as previously described [21].

**Table 1:** Characteristics of SpA patients

	SpA patients
Total number	56
Age (mean ± SD)	46 ± 16
Male/female	32 (57%)/24
Known duration of the SpA :	46 (82%)
Average (min-max)	6 years (2 weeks–30 years)
Known HLAB27 status :	39 (70%)
HLAB27 positive/negative	23 (59%)/16
Known therapeutic treatment :	53 (95%)
Without therapeutic treatment	13 (25%)
Treated with NSAIDs	22 (42%)
Treated with corticosteroids	26 (49%)
Treated with DMARDs	27 (51%)
Including anti-TNF-α biotherapies	10 (19%)

*NSAIDs* nonsteroidal anti-inflammatory drugs, *DMARDs* disease-modifying antirheumatic drugs

### Organotypic culture

An organotypic model of mouse ankle was used to study the enthesis of Achilles tendon insertion into the calcaneus bone, as previously described [22]. Samples were retrieved from 12-week-old DBA/2 male mice. After 1 day of rest in serum-free Dulbecco’s Modification of Eagle Medium (DMEM), the ankles were treated for 3 days with a cocktail of pro-inflammatory cytokines: TNF-α, IL-22, IL-17, IL-6, and IL-1β (each at 10 ng/mL). While one leg was cytokine-stimulated, the contralateral one served as the untreated control.

### Immunohistochemistry

Mouse ankles were isolated from 12-week-old DBA/2 male animals and fixed in acidified formal alcohol (AFA) for 24 h at room temperature. Tissue samples were paraffin-embedded after decalcification and dehydration and sliced with a microtome. The tissue sections were incubated overnight with anti-phospho-Smad5 primary antibody (ABcam ab92698). Sections were revealed by the use of the HRP Envision™ anti-rabbit kit and tetrahydrochloridediaminobenzidine (DAB) staining (Dako). Sections were slightly counter-stained with Mayer’s hematoxylin staining.

### Entheseal cell primary culture

Primary osteoblasts, chondrocytes, and tenocytes were isolated from newborn (4–6 days) SWISS mice by successive enzymatic digestion of calvaria, knee, and femoral head cartilage and tail tendon, respectively [23]. Tenocytes and chondrocytes were grown 1 week in DMEM supplemented with 10% of serum, penicillin (10 U/mg), and streptomycin (0.1 mg/mL). Tenocytes were passed once but not chondrocytes. Osteoblasts were grown for 3 weeks in the same medium, but ascorbic acid (50 μg/mL) was added from day 3 after seeding and β-glycerophosphate (10 mM) from day 10. Convenient osteoblast maturation in these conditions has already been validated [23].

### Stretch and cytokine challenges of cultured cells

Chondrocytes and osteoblasts were placed in serum-free conditions for 24 h, with 0.1% bovine serum albumin, before any mechanical or cytokine stimulation. For tenocyte cultures, serum content was reduced to 1%. Cells were subjected to equibiaxial stretching using Flexcell® Tension Plus FX-3000 (Flexcell® International) for 2 or 6 h (0.5% Hz, 4.5% elongation). Inflammatory stress was induced by addition of recombinant mouse cytokines (Immunotools) for 6 or 24 h: 10 ng/mL of TNF-α and 100 ng/mL of IL-17 (also known as IL-17A) or IL-22.

### Cell treatments with BMP-2, -4, and Noggin

Chondrocytes and tenocytes were grown for 1 week as described before. They were then treated for the next 10 days in DMEM containing serum (10%) and ascorbic acid (50 μg/mL), with or without recombinant human BMP-2 or -4 (20, 100, or 250 ng/mL) or 100 ng/mL of recombinant mouse Noggin (Immunotools).

### ELISA analysis

All ELISA experiments were performed according to the manufacturers’ instructions. ELISA kits for BMP-2, -4, and -7 and osteoprotegerin (OPG) were purchased from R&D Systems, and kits for prostaglandin E_2_ (PGE_2_) and IL-6 from Cayman, Invitrogen.

### Alkaline phosphatase (AP) specific activity

AP activity was measured using p-nitrophenyl phosphate as a substrate as described before [22]. For standardization, proteins were quantified by BCA assay kit (Pierce).

### Gene expression analysis

Total RNA extraction, reverse transcription, and real-time QPCR were realized as previously described [22]. Glyceraldehyde-3-phosphate dehydrogenase (*Gapdh*) was used as a reference gene in cultured cells. For normalization of tissue samples, the geometric average of three housekeeping genes was calculated: β-actin (*Actb*), hypoxanthine-guanine phosphoribosyltransferase (*Hprt*), and ribosomal Protein L13a (*Rpl13a*).

### Statistical analysis

For the analysis of human samples, data from OA patients were compared to SpA patients by the use of unpaired *T* test Welch corrected. Linear correlations were established with Pearson’s test. For the analysis of mouse samples, at least 4 independent experiments were performed. Data were analyzed by the use of two-sided Mann-Whitney tests. Statistical significance was defined as *p* < 0.05 (*).

## Results

### In synovial fluid of SpA patients, BMP-2 content is higher and BMP-4 is lower than in OA samples

**Fig. 1:**
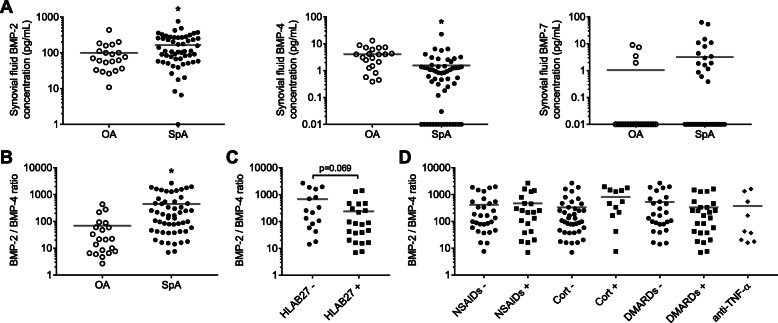
Levels of BMP-2, -4, and -7 in synovial fluids from patients with peripheral spondyloarthritis (SpA), compared to osteoarthritic (OA) patients. **a** BMP-2, BMP-4, and BMP-7 contents were measured by ELISA. **b** BMP-2/BMP-4 ratio was calculated to illustrate the equilibrium between the two growth factors. *Statistical difference between the *n* = 56 SpA and *n* = 21 OA samples. Among SpA patients, BMP-2/BMP-4 ratio was compared between HLAB27-positive and -negative status (**c**), and patients treated (+) or not (−) with NSAIDs, corticosteroids (Cort), DMARDs, or anti-TNF-α biotherapies (**d**). Symbols represent individual data points; the stick band represents the mean of each group

BMP-2, -4, and -7 concentrations were measured by ELISA in the synovial fluid (SF) of 56 SpA patients (Fig. 1a). BMP levels were independent of age and sex of the patients (data not shown). Compared to OA patients (*n* = 21), BMP-2 content was particularly elevated in SpA SF (165 ± 19 versus 100 ± 21 pg/mL; mean ± SEM, *p* = 0.025). In opposition, BMP-4 concentration was lower in SpA SF than in OA samples (1.6 ± 0.4 versus 4.1 ± 0.7 pg/mL, *p* = 0.004). In consequence, the balance between BMP-2 and -4 was drastically impaired in the SF of SpA patients, as clarified by the calculation of BMP-2/BMP-4 ratio, which is increased by 6.5 folds (Fig. 1b, *p* < 0.001). BMP-7 was detected in only 16 SpA SF (28%) and in only 4 OA SF (19%) (Fig. 1a). Neither BMP-2 nor BMP-4 content seemed significantly affected by HLAB27 status of the SpA patients (Suppl. figure 1). Nevertheless, BMP-2/BMP-4 ratio tended to be higher in HLAB27-negative patients (660 ± 203 versus 237 ± 80 for HLAB27-positive patients, *p* = 0.069, Fig. 1c). However, these results should be confirmed on a larger cohort, as only 39 patients were included. In addition, multiple comparison tests revealed no effect of treatments as regards the following groups: ± therapeutic treatment (data not shown), ± NSAID, ± corticosteroid, ± DMARD, and ± anti-TNF-α (Fig. 1d). But again, these results should be completed on a larger cohort.

### BMP levels in SF of SpA patients correlate OPG and inflammatory markers

**Fig. 2:**
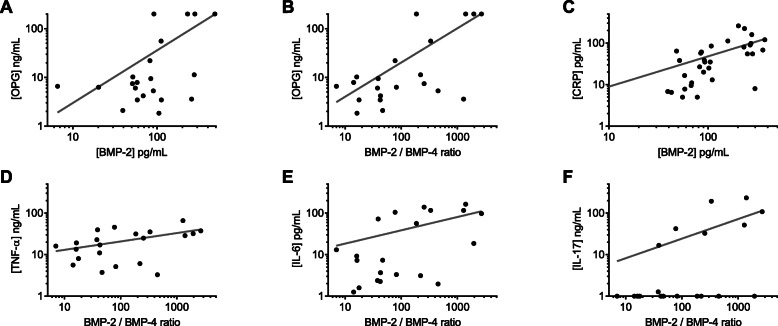
Correlations between BMP-2 levels or BMP-2/BMP-4 ratios and inflammatory markers for SpA patients. CRP concentration (**c**) was determined in *n* = 34 SpA patients and OPG, TNF-α, IL-6, and IL-17 in *n* = 21 patients (**a**, **b**, **d–f**). Correlations were examined relative to BMP-2 levels (**a**, **c**) or to the BMP-2/BMP-4 ratios (**b**, **d–f**). Symbols represent individual data points; the plain line represents the linear regression

Because of the osteogenic potential of BMPs, a correlation between BMP-2 and -4 contents in the SF of SpA patients and its level of osteoprotegerin (OPG), which is a bone anti-resorptive marker, was expected. BMP-2 concentration indeed correlated OPG levels. Interestingly, an even stronger correlation was observed between OPG and the BMP-2/BMP-4 ratio (Table 2, Fig. 2a, b). Besides, we were also concerned by any connection between BMP levels and the inflammatory status of the patients (Table 2, Fig. 2c–f). C-reactive protein (CRP) concentration is the most widespread serum inflammatory marker used in the clinical management of SpA. Again, a correlation was observed between BMP-2 and CRP levels (Fig. 2c). However, CRP content did not correlate neither BMP-4 concentration directly (data not shown), nor BMP-2/BMP-4 ratio (Table 2). In the same vein, correlations were investigated between the SF’s pro-inflammatory cytokines TNF-α, IL-6, and IL-17 and BMP levels (Table 2). TNF-α and IL-6 content correlated the BMP2/BMP-4 ratio (Fig. 2d, e) but not BMP-2 level. IL-17 was detected in only 8 SpA SF over 21 (38%), yet a positive correlation was identified with BMP-2/BMP-4 ratio (Fig. 2f).

**Table 2:** Correlation analysis between inflammatory markers and BMP-2 levels and BMP-2/BMP-4 ratios in SF of SpA patients

	*n*	OPG	CRP	TNF-α	IL-6	IL-17
		21	33	21	21	21
	Mean ± SEM	46.0 ± 10.4 ng/mL	58.0 ± 10.4 pg/mL	22.5 ± 3.6 ng/mL	44.9 ± 12.0 pg/mL	32.9 ± 14.3 pg/mL
BMP-2 levels	*R*^2^	0.395^#^	0.264^#^	0.179	0.175	0.075^Ɨ^
	*p* value	0.002*	0.002*	0.056	0.060	
BMP-2/BMP-4 ratio	*R*^2^	0.551^#^	0.041	0.225^#^	0.196	0.018^Ɨ^*
	*p* value	0.0001*	0.260	0.030*	0.044*	

*Significant *p* values

^#^*R*^2^ > 0.2

^Ɨ^Non-parametric statistical analysis using the Spearman test

### Cytokines regulate BMP expression in entheseal cells

**Fig. 3:**
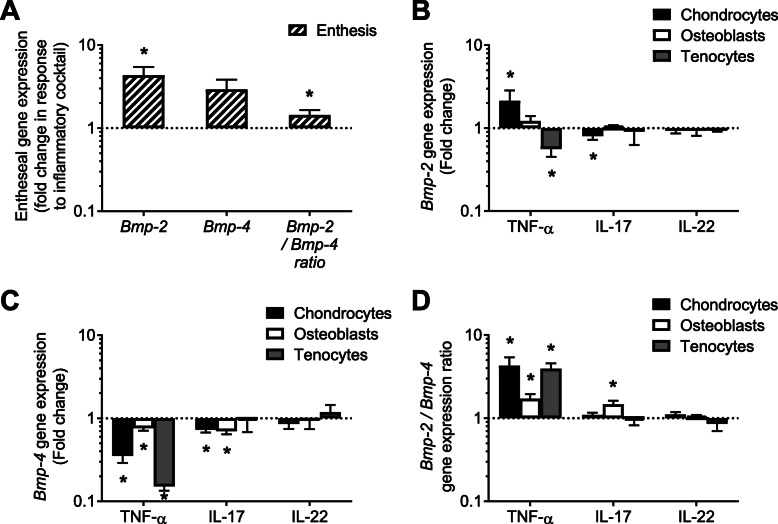
Regulation of *Bmp-2* and *-4* gene expression in response to cytokine stimulation. **a** Total RNA was extracted from mouse ankle enthesis after organotypic culture, for real-time PCR analysis. The explants were stimulated 72 h by a cocktail of pro-inflammatory cytokines (TNF-α, IL-22, IL-17, IL-6 and IL-1β, 10 ng/mL each). Gene expression of *Bmp-2* and *-4* and the *Bmp-2*/*Bmp-4* ratio were compared to the contralateral non-treated samples. **b–d** Cultured chondrocytes (black bars), osteoblasts (white bars), and tenocytes (gray bars) were treated 24 h by either TNF-α (10 ng/mL), IL-17 (100 ng/mL), or IL-22 (100 ng/mL). Gene expression of *Bmp-2* (**b**) and *-4* (**c**) and the *Bmp-2*/*Bmp-4* ratio (**d**) were compared to non-treated control cells

Our organotypic model of mouse ankle was used to investigate whether BMP expression was sensitive to cytokines within the enthesis of Achilles tendon (Fig. 3a). Addition of an inflammatory cocktail containing TNF-α, IL-22, IL-17, IL-6, and IL-1β increased *Bmp-2* gene expression (4 folds), tended to augment *Bmp-4*, and did not modify *Bmp-7* (data not shown). Thus, a little imbalance of the *Bmp-2/Bmp-4* ratio in favor of *Bmp-2* was also observed in this model (1.5 folds, Fig. 3a). To determine which cells in the enthesis might produce BMPs, mouse primary chondrocytes, mature osteoblasts, or tenocytes were treated with the cytokines that are typically suggested to be involved in SpA pathogenesis [24, 25]: TNF-α, IL-17, and IL-22, separately. The more noticeable effects were observed in response to TNF-α: *Bmp-2* gene expression was increased in chondrocytes and decreased in tenocytes, *Bmp-4* was diminished in all three cell types (Fig. 3b, c), and *Bmp-7* was decreased in chondrocytes (data not shown). IL-17 had a similar effect as TNF-α on *Bmp-4* mRNA, but to a lesser extent, and it induced a slight reduction of *Bmp-2* mRNA in chondrocytes. IL-22 had no visible effects (Fig. 3b, c). It appears that wherever *Bmp-2* and/or *Bmp-4* expression was regulated, the balance always tilted in favor of *Bmp-2* (Fig. 3d). A discrepancy could be noted in the regulation of *Bmp-4* gene expression between the organotypic model and the cell culture, even if the *Bmp-2/Bmp-4* ratio was in favor of *Bmp-2* in both cases.

### Stretch also regulates BMP expression in entheseal cells

**Fig. 4:**
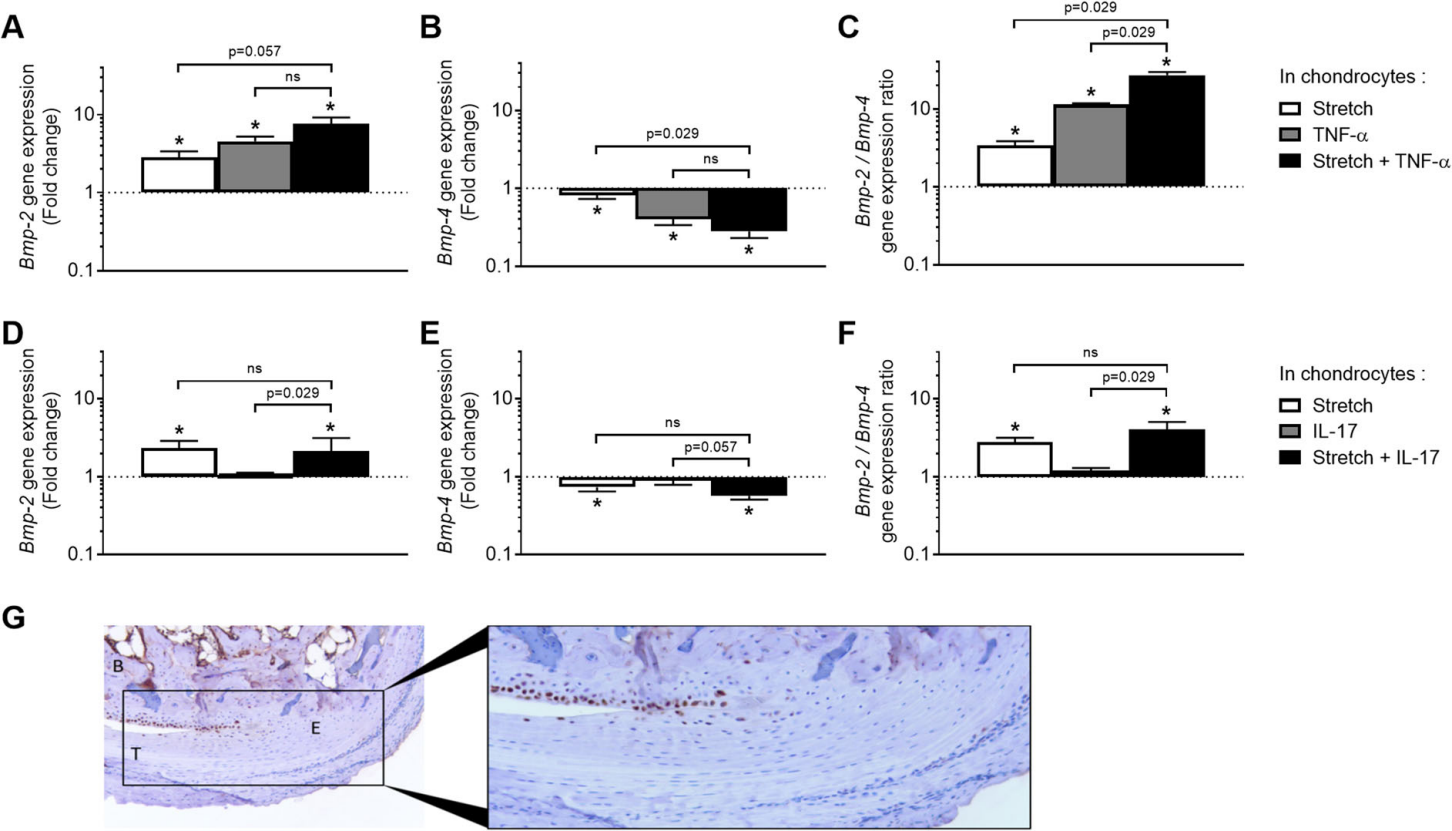
Regulation of *Bmp-2* and *-4* gene expression and phospho-smad5 in response to mechanical stimulation. **a–c** Dynamic stretch was applied for 2 h or 6 h to cultured chondrocytes (black bars), osteoblasts (white bars), and tenocytes (gray bars). Gene expression of *Bmp-2* (**a**) and *-4* (**b**) and the *Bmp-2*/*Bmp-4* ratio (**c**) were compared to non-stretched control cells. **d–f** Cultured chondrocytes were stimulated 6 h by stretch (white bars), or 10 ng/mL of TNF-α (gray bars) separately or simultaneously (black bars). Gene expression of *Bmp-2* (**d**) and *-4* (**e**) and the *Bmp-2*/*Bmp-4* ratio (**f**) were compared to non-stretched untreated control cells. **g** Immunostaining of phospho-smad5 in sane mouse ankle sections. The section comprises the enthesis -*E-* of the Achilles tendon -*T-* insertion into the calcaneus bone -*B-*. Some chondrocytes of the sesamoid cartilage zone were positive, as revealed by an intracellular brown staining. Sections were slightly counter-stained with Mayer’s hematoxylin staining. *p < 0.05 in comparison to the control resting condition; *p* values are indicated for the differences between the other conditions, ns: not significant (*p* > 0.1)

Chondrocytes, osteoblasts, or tenocytes in monolayer culture were subjected to 2 h or 6 h of a cyclic stretch to assess the effects of mechanical stimulation on BMP expression (Fig. 4a-c). Concerning *Bmp-2* gene expression, the strongest result was a stretch-induced upregulation in chondrocytes (Fig. 4a). *Bmp-4* mRNA was decreased in all three cell types by 6 h of mechanical stress (Fig. 4b). *Bmp-2*/*Bmp-4* ratio was thus upregulated, especially in chondrocytes (Fig. 4c). *Bmp-7* gene expression was not detected in tenocytes and almost not regulated in osteoblasts and chondrocytes (data not shown). Because chondrocytes were the more responsive to cytokines (Fig. 3) and stretch (Fig. 4a–c), we focused on this cell type to evaluate the effect of the combination of 6-h long dynamic stretch and TNF-α. The stretch- and TNF-α-induced upregulation of *Bmp-2* and downregulation of *Bmp-4* expression were additive (Fig. 4d, e). *Bmp-2*/*Bmp-4* ratio was calculated, in order to provide an overview of the equilibrium regulation. Stretch and TNF-α alone induced a 3-fold and a 10-fold increase, respectively. The combination of both stimulations led to a rise of around 30 folds in the expression of *Bmp-2* relative to *Bmp-4* (Fig. 4f). By the use of a similar experiment, the response to the combination of stretch and IL-17 was assessed: addition of IL-17 did not amplify the effect of the stretch alone (data not shown).

### Mechanical stress may activate BMP pathway in situ

Activation of the BMP canonical pathway was revealed by the immunostaining of a phosphorylated form of Smad5 (phospho S463 and S465) on mouse ankle sections (Fig. 4g). Some chondrocytes were positive, but no tenocytes of the Achilles tendon or osteocytes of the underlying bone. Interestingly, phospho-Smad5 was selectively detected in chondrocytes of the sesamoid cartilage zone, which is a location of high mechanical stress [26]. This cell-specificity suggests that the steady-state activation of BMP signaling is already high in mechanically stimulated cartilage cells nearby the enthesis.

### The response to 6 h of mechanical stress is independent of IL-6 and PGE_2_

**Fig. 5:**
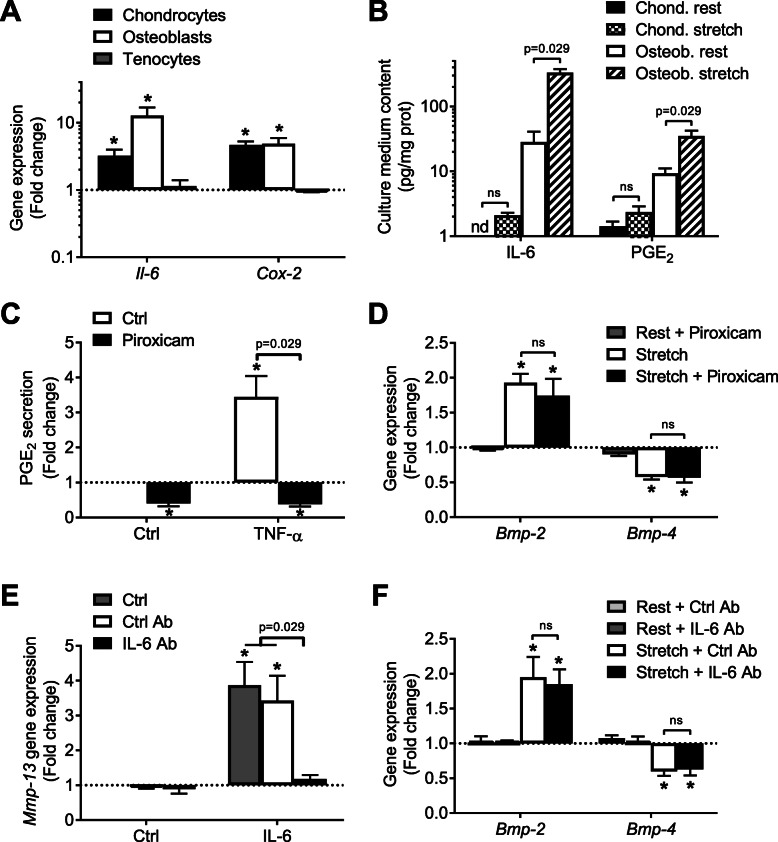
Connection between IL-6 and PGE_2_ inflammation pathways and the mechanical stimulation. **a** Dynamic stretch was applied for 6 h to cultured chondrocytes (black bars), osteoblasts (white bars), and tenocytes (gray bars). Gene expression of *Il-6* and *Cox-2* were compared to non-stretched control cells. **b** IL-6 and PGE_2_ secretion was analyzed in the culture medium of chondrocytes and osteoblasts either in control (rest) or stretched conditions. **c**, **d** Piroxicam was added to chondrocytes to inhibit PGE_2_ synthesis. **c** The piroxicam-induced reduction in PGE_2_ content of the culture medium was validated in TNF-α-treated chondrocytes. **d** The effect of piroxicam on gene expression of *Bmp-2* and *-4* was analyzed in control or stretched conditions. **e**, **f** IL-6 blocking antibodies (Ab) were added to chondrocytes to inhibit IL-6 pathway. The effect of this treatment was compared to non-specific IgG1 antibodies (ctrl Ab). **e** *Mmp-13* gene expression was analyzed in control or IL-6-treated chondrocytes and (**f**) *Bmp-2* and *-4* gene expression in control (rest) or stretched chondrocytes. *p < 0.05 in comparison to the control condition, *p* values are indicated for the differences between the other conditions, ns: not significant (*p* > 0.1)

Overproduction of pro-inflammatory mediators has already been shown in cultured chondrocytes and osteoblasts in response to mechanical stress, hence we investigated IL-6 and prostaglandin E_2_ (PGE_2_) production in our stretching model [27, 28]. The stretch did induce an inflammatory response in osteoblasts and chondrocytes (Fig. 5a, b). *Il-6* gene expression was markedly induced by 6 h of stretch in both cell types, but not in tenocytes (Fig. 5a), and IL-6 secretion by osteoblasts rapidly increased in accordance (Fig. 5b). In addition, in chondrocytes and osteoblasts, cyclooxygenase-2 (*Cox-2*) expression was upregulated and an increased release of its enzymatic product PGE_2_ was observed. Like IL-6, levels of released PGE_2_ were neatly higher in osteoblast cultures than in chondrocytes (Fig. 5b). By the way, very little *TNF-α* gene expression was detected in all the three cell types and it was not induced in response to mechanical stress (data not shown).

Next, we wondered if, in cultured chondrocytes, the stretch-induced *Bmp-2*/*Bmp-4* mRNA deregulation was mediated by the inflammation. Piroxicam was used as NSAID to inhibit Cox activity, which was validated by a dramatic decrease in TNF-α-induced PGE_2_ production (Fig. 5c). Piroxicam addition had no effect on *Bmp-2* or *-4* mRNA in control conditions and it did not alter the stretch-induced *Bmp-2* or *-4* regulations (Fig. 5d). On the other hand, IL-6 blocking antibodies were used to assess the possible role of autocrine IL-6 in the stretch-mediated regulations. IL-6-induced *Mmp-13* stimulation was used to check the efficiency of the blockage of IL-6 action (inhibition by 95%, Fig. 5e). Similarly, addition of IL-6 blocking antibodies had no detectable consequences on *Bmp-2* or *-4* gene expression (Fig. 5f). Of note, *Il-6* mRNA was logically induced by TNF-α in chondrocytes (7-fold change), but IL-6 blockage had no effect on TNF-α-induced BMP regulation (Suppl. figure 2).

### Osteogenic effects of BMP-2 and BMP-4 on chondrocytes and tenocytes

**Fig. 6:**
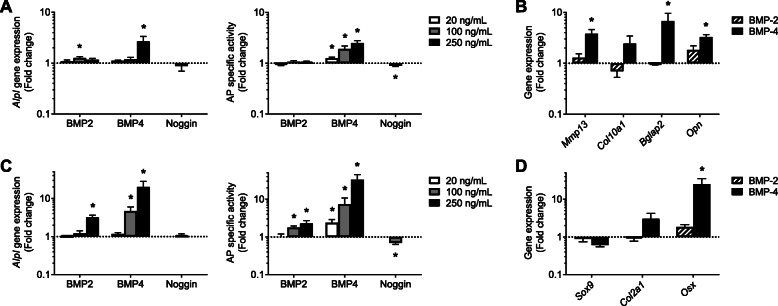
Osteoinductive effects of BMP-2 and -4 on cultured chondrocytes and tenocytes. BMP-2 or 4 (20, 100 or 250 ng/mL) or Noggin (100 ng/mL) were added during 10 days to cultured chondrocytes (**a**, **b**) and tenocytes (**c**, **d**). Alkaline phosphatase gene expression (*Alpl*) and specific activity were compared to untreated control cells (**a**, **c**). Additional differentiation marker genes were analyzed in cultured cells treated at the highest dose (**b**, **d**): matrix metalloproteinase 13 (*Mmp13*), type II and X collagen (*Col2a1*, *Col10a1*), bone γ-carboxyglutamate protein 2 (*Bglap2*, coding osteocalcin), osteopontin (*Opn*), SRY-box transcription factor 9 (*Sox9*), and osterix (*Osx*)

First, the steady-state level of some osteoblast and chondrocyte markers was used to characterize our different cell cultures: osteocalcin (or bone γ-carboxyglutamate protein 2, *Bglap2*) and tissue-nonspecific alkaline phosphatase (*Alpl*) gene expression, the ratio of type II to type I collagen (*Col2a1*/*Col1a1*) mRNA, and alkaline phosphatase (AP) specific activity. The latter was 5 folds higher in chondrocytes than in tenocytes, and again 50 folds higher in mature osteoblasts than in chondrocytes (Suppl. figure 3). In cultured chondrocytes, BMP-4 slightly enhanced AP specific activity and gene expression (Fig. 6a). The BMP antagonist Noggin was able to reduce AP activity (Fig. 6a); however, its effect was very faint, probably because the basic level of BMPs in the culture medium was low. Additional genes were investigated to better characterize the BMP-induced maturation of chondrocytes as those cells may hypertrophy and/or trans-differentiate into osteoblasts [29]. Matrix metalloproteinase 13 (*Mmp-13*) and type X collagen (*Col10a1*) were used as hypertrophy and early trans-differentiation markers, and *Bglap2* and Osteopontin *(Opn)* as later markers. The BMP-4-induced upregulation of *Alpl* was associated with an increase in *Mmp13*, *Bglap2*, and *Opn* mRNAs, at the highest dose (Fig. 6b). In parallel, BMP-2, and more neatly BMP-4, enhanced AP-specific activity and gene expression in cultured tenocytes (Fig. 6c). Again, Noggin was able to faintly reduce AP activity (Fig. 6c). Because tendon cells are prone to trans-differentiate either into the chondrocyte or the osteoblast lineage [30], both chondrocyte (*Sox9* and *Col2a1*) and osteoblast markers (Osterix, *Osx*) were further explored (Fig. 6d). *Sox9* and *Col2a1* mRNA were not modulated, neither in the presence of BMP-2 nor of BMP-4. On the contrary, *Osx* was strongly increased by BMP-4, indicating that tenocytes rather trans-differentiated in osteoblast-like than in chondrocyte-like cells (Fig. 6d). Globally, in our models, BMP-4 seemed to be more potent than BMP-2. Indeed, BMP-4 treatment augmented AP in cultured chondrocytes and in tenocytes, and AP induction was higher in response to BMP-4, as compared to the same doses of BMP-2.

## Discussion

Primarily, we measured an enhanced BMP-2/BMP-4 ratio in SF from patients with SpA. BMP deregulation has already been observed in SpA patients’ serum; however, to our knowledge, this is the first study assessing BMP levels in SpA patients’ SF. The consistency of our results was validated with respect to previous studies that assessed BMP contents in SF from OA patients [31–34]. Three independent studies have demonstrated that BMP-2 serum concentration was in the range of 100 pg/mL and neatly higher in ankylosing spondylitis patients than in healthy controls [14, 15, 35]. Our result showing that BMP-2 content is particularly elevated in SpA SF seems thus coherent, especially because a correlation between serum and SF BMP-2 levels was expected [31]. In accordance, an increase in BMP-2 protein expression has already been detected in the inflamed synovial tissue of SpA patients [36]. Of note, BMP-2 seems almost absent from the healthy synovium, tendon, or articular cartilage [36–38], but its expression drastically increases in fibroblasts, chondrocytes, and osteoblasts during OA osteophyte formation [37, 39], as well as in fibroblasts and chondrocytes during ossification of various ligaments [38]. In addition to the previously observed correlation of BMP-2 levels with the Bath Ankylosing Spondylitis Indexes [14, 15], we noticed a correlation with OPG and CRP markers. Altogether, these data support the clinical relevance of BMP-2 assessment. In contrast, the drop of BMP-4 levels in SpA SF was a particularly striking result. Indeed, BMP-4 serum content has been shown to be either equal or superior in SpA patients than in healthy donors [14, 15, 35]. However, a decrease in BMP-4 expression has already been observed in synovial tissue of patients with OA and rheumatoid arthritis [40]. Our results settle that BMP factors are deregulated locally in the SpA joint. Yet, one of the limitations of this study is that it did not determine whether such deregulation was also systemic. Nevertheless, our data suggest an unsuspected role of BMP-4 modulation in SpA.

In addition, the absence of HLAB27 seemed to emphasize the unbalance of BMP-2/BMP-4 ratio in the SF of SpA patients (*n* = 39). This trend might have a clinical relevance as among ankylosing spondylitis patients, HLAB27-negative ones present a higher prevalence of peripheral arthritis [41]. Besides, it has recently been revealed that HLAB27 most likely crosstalks with BMP signaling in immune cells from SpA patients [42]. The mechanism for HLAB27 involvement in BMP production has not been addressed in this study. On the other hand, the participation of BMP pathway in the development of some of the characteristic features of SpA has also been stated in the absence of HLAB27 [6]. More generally, the molecular action of HLAB27 in the pathophysiology of SpA remains unclear.

Our data in cultured entheseal cells nicely paralleled our results in patients, since any stimuli mimicking local mechanical stress and inflammation steadily unbalanced the BMP-2/BMP-4 equilibrium, in favor of BMP-2. The imbalance was especially obvious when the combination of TNF-α and cyclical stretch was applied to chondrocytes. In these cells, the molecular mechanisms of BMP-2 induction by TNF-α have already been characterized, involving both transcriptional upregulation and mRNA stabilization [43]. Yet, inflammatory cytokines and BMP-2 have opposing effects regarding osteogenic differentiation, so the consequences of their concurrent presence in the enthesis regarding bone formation are uncertain [44–46]. In addition, the importance of the cross-talk between BMPs and inflammatory pathways should not be overlooked [47]. Not only pro-inflammatory cytokines can modulate the production of BMPs but also the BMPs can interfere with their effects. For instance, blockade of BMP pathway potentiates IL-17 and TNF-α effects in rheumatoid synoviocytes [48]. As the various BMP members have been suggested to exert both pro- and anti-inflammatory actions (see [49] for review), any unbalance in BMP signaling may disturb the inflammation process. However, one can speculate that once the inflammation is resolved, the osteogenic properties of BMPs could take the lead. Our results also suggest that mechanical stress might contribute to local inflammation. The production of pro-inflammatory factors, including IL-6 and PGE_2_, by mechanosensitive cells may participate in the enthesitis process. Especially, the osteoblasts and osteocytes of the underlying bone may be a source of soluble factors that could act through the entire enthesis structure.

Our more unexpected result is the decrease in BMP-4 that conversely corresponds to the increase in BMP-2. Phylogenetic analysis reveals that among the 20 members, BMP-2 and -4 are so analogous that they form alone a subgroup [50, 51]. BMPs mainly trigger cellular responses through six different type I and type II receptors activating the canonical Smad pathway [50, 51]. Nonetheless, single receptor knock-down experiment suggests very similar signaling mechanisms for BMP-2- and BMP-4-induced osteogenic effect [52]. Further increasing the complexity of BMP pathway, mature ligands are secreted as disulfide-linked dimers, offering numerous possible combinations that include BMP-2 or -4 [53]. Both BMP-2 and -4 promoters share common regulatory elements, including binding sites for the master bone and cartilage transcription factors Runx-2 and Sox-9, respectively [54, 55]. Interestingly, two putative NF-κB response elements were found in the *BMP-2* gene, whereas *BMP-4* one contains none [56]. In agreement, NF-κB has been shown to mediate the TNF-α-induced augmentation of *BMP-2* gene expression in chondrocytes [43, 57].

As members of the large BMP family, both BMP-2 and -4 have osteoinductive properties [52, 58, 59]. The quality and magnitude of BMP-2 and -4 effects on human mesenchymal stem cells (MSCs) appear comparable [52]. During arthritis, MSCs coming from the bone marrow can invade the soft tissues of the enthesis, in addition to local progenitor cells [60]. Such a set of multipotent cells could not only be involved in the immunoregulation process, but also be a major source of osteoblasts, contributing to the SpA ossification process [60]. Of interest, MSCs from SpA patients have enhanced osteogenic differentiation ability as compared with MSCs of healthy donors, and this greater osteogenic potential is especially due to BMP-2 overexpression [61]. In our study, both BMP-2 and BMP-4 ligands were able to induce osteoblastic-like differentiation in chondrocytes and tenocytes, indicating that BMPs are not only effective on progenitors, but also on already differentiated skeletal cells. Surprisingly, we observed a stronger osteoinductive effects with BMP-4 than with BMP-2. Indeed, such a differential does not match with the shift in favor of BMP-2 that we surmise to be involved in SpA ossification. More refined investigations are required, in particular using different combinations of BMP-2/BMP-4 dimers, to clarify that point. The expression of bone formation markers in chondrocytes was expected, but was more startling in tenocytes. Yet, the capacity of BMP-2 to favor osteogenic differentiation in tendon cells has already been suggested [12, 62, 63]. In agreement with our results, in one of these studies, BMP-2 was increased by mechanical stimulation, and in another one, by PGE_2_-mediated inflammation [12, 62]. Stretch-induced BMP-4, alike BMP-2, was also observed in tendon cells [64]; however, to our knowledge, no study has demonstrated its osteogenic potential in these cells. Altogether, a local increase in BMPs might modify the mineralizing capacity of entheseal cells to favor ossification in SpA joint.

Although all BMPs are extremely similar, they are rarely redundant. We believe that the switch of BMP-2/BMP-4 equilibrium that we observed is of particular importance. Deficiency in BMP-2, as well as in BMP-4, both potent bone anabolic factors, is embryonically lethal. However, conditional gene targeting suggests slight functional differences between one and the other (see [50] for review). Focusing on the enthesis structure, BMP-4 has been identified as a key component for bone ridge formation [65, 66]. Actually, the transcription factor scleraxis regulates BMP-4 in tendon cells to induce cartilage formation at the tendon-skeleton junction [65]. In these bony structures, the progenitors are not chondrocytes but rather a distinct pool of tenocyte-like cells [66]. Adding to our results, the involvement of BMP-4 in bone formation during enthesis development further justifies the exploration of its role in pathologic ossification in the context of SpA.

## Conclusions

More investigations are needed to decipher how an increased BMP-2 and a decreased BMP-4 level could interfere with the abnormal ossification of the enthesis in SpA. Exploring experimental SpA models would certainly supply more information on the impact of BMP-2/BMP-4 imbalance in this specific ankylosis. Identification of the BMP members that may stimulate the first steps of enthesis ossification in SpA could provide promising therapeutic targets. Disease and tissue context probably regulate very finely BMP signaling so that increasing both specificity and effectiveness of BMP-targeting strategies could be mandatory [67]. Nevertheless, our results settled the interest of investigating BMP pathways in SpA patients.

## Supplementary information

**Additional file 1: Supplementary figure 1.** Levels of BMP-2 (A) and -4 (B) in synovial fluids from patients with peripheral spondyloarthritis (SpA). 23 HLAB27-positive SpA patients were compared to 16 HLAB27-negative ones.

**Additional file 2: Supplementary figure 2.** Connection between IL-6 and PGE_2_ inflammation pathways and the TNF-α stimulation. TNF-α-treated cultured chondrocytes (10 ng/mL, 24 h) were compared to control cells. (A) Gene expression of *Il-6* and *Cox-2* were analyzed. (B) IL-6 blocking antibodies (Ab) were added to inhibit IL-6 pathway. The effect of this treatment was compared to non-specific IgG1 antibodies (ctrl Ab). *Bmp-2* and *-4* gene expression were analyzed. ns: not significant (*p*>0.1).

**Additional file 3: Supplementary figure 3.** Comparison of the steady-state level of some osteogenic and chondrogenic markers between cultured osteoblasts, chondrocytes and tenocytes. (A) *Bglap2*, *Alpl*, *Col1a1* and *Col2a1* relative gene expressions and (B) alkaline phosphatase (AP) specific activity were analyzed in cultured chondrocytes (black bars), osteoblasts (white bars) and tenocytes (grey bars). Data from osteoblasts and tenocytes were compared to chondrocytes (*) and data from chondrocytes and tenocytes were compared to osteoblasts (^#^).

## Data Availability

The datasets used and/or analyzed during the current study are available from the corresponding author on reasonable request.

## Abbreviations

BMP: Bone morphogenetic protein
CRP: C-reactive protein
DMARDs: Disease-modifying antirheumatic drugs
IL: Interleukin
NSAIDs: Nonsteroidal anti-inflammatory drugs
OA: Osteoarthritis
PGE_2_: Prostaglandin E_2_
SF: Synovial fluid
SpA: Peripheral spondyloarthritis
