# Supplementary figures and images for: Enhanced BMP-2/BMP-4 ratio in patients with peripheral spondyloarthritis and in cytokine- and stretch-stimulated mouse chondrocytes
Source: Arthritis Res Ther. 2020 Oct 12;22:234. doi: 10.1186/s13075-020-02330-9 (PMC7552569; doi:10.1186/s13075-020-02330-9)

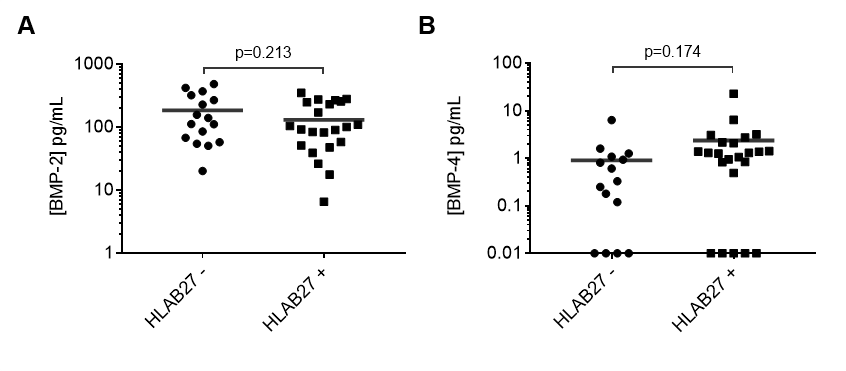

Supplement: Supplementary file 1 — Additional file 1: Supplementary figure 1. Levels of BMP-2 (A) and -4 (B) in synovial fluids from patients with peripheral spondyloarthritis (SpA). 23 HLAB27-positive SpA patients were compared to 16 HLAB27-negative ones. [file 13075_2020_2330_MOESM1_ESM.tif]

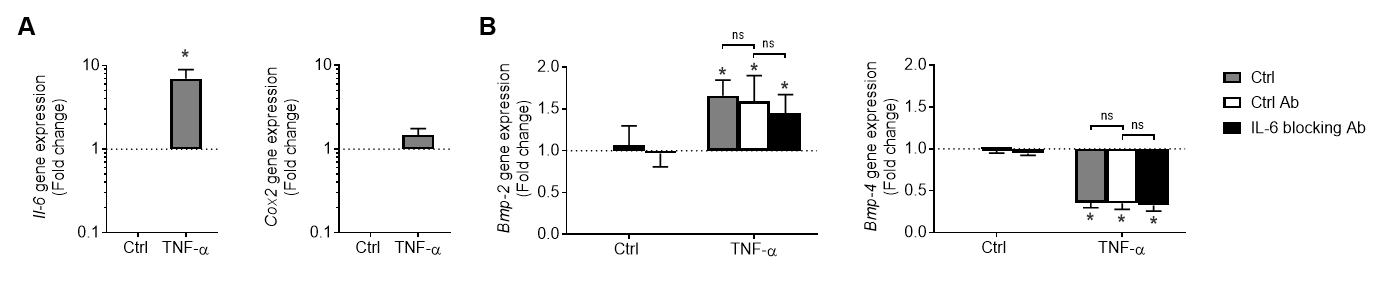

Supplement: Supplementary file 2 — Additional file 2: Supplementary figure 2. Connection between IL-6 and PGE2 inflammation pathways and the TNF-α stimulation. TNF-α-treated cultured chondrocytes (10 ng/mL, 24 h) were compared to control cells. (A) Gene expression of Il-6 and Cox-2 were analyzed. (B) IL-6 blocking antibodies (Ab) were added to inhibit IL-6 pathway. The effect of this treatment was compared to non-specific IgG1 antibodies (ctrl Ab). Bmp-2 and -4 gene expression were analyzed. ns: not significant (p>0.1). [file 13075_2020_2330_MOESM2_ESM.tif]

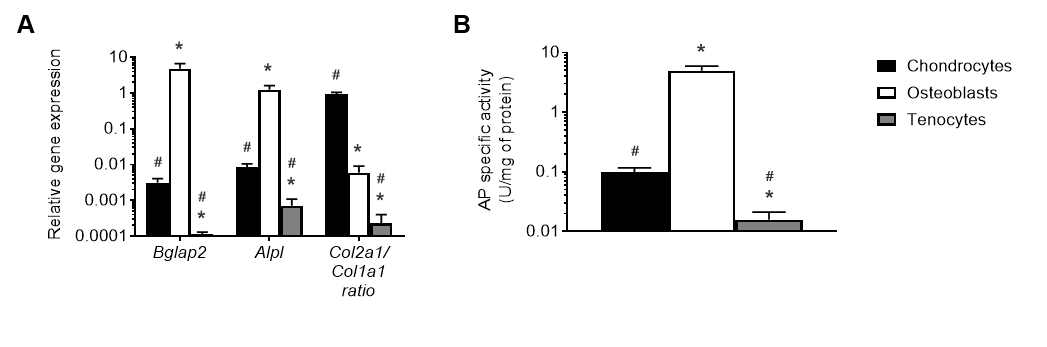

Supplement: Supplementary file 3 — Additional file 3: Supplementary figure 3. Comparison of the steady-state level of some osteogenic and chondrogenic markers between cultured osteoblasts, chondrocytes and tenocytes. (A) Bglap2, Alpl, Col1a1 and Col2a1 relative gene expressions and (B) alkaline phosphatase (AP) specific activity were analyzed in cultured chondrocytes (black bars), osteoblasts (white bars) and tenocytes (grey bars). Data from osteoblasts and tenocytes were compared to chondrocytes (*) and data from chondrocytes and tenocytes were compared to osteoblasts (#). [file 13075_2020_2330_MOESM3_ESM.tif]
